# Extremely Late Local Recurrence of a Choroidal Melanoma, 51 Years Following Cobalt-60 Plaque Radiation Therapy

**DOI:** 10.1055/a-2793-7860

**Published:** 2026-03-17

**Authors:** Gabriel Damien Verdon, Ann Schalenbourg, Alessia Pica

**Affiliations:** 1 Ophthalmology – FAA – University of Lausanne, University Eye Hospital Jules Gonin, Lausanne, Switzerland; 2 Center for Proton Therapy – ETH Domain, Paul Scherrer Institut, Villigen, Switzerland

## Background

Conservative radiation therapy of uveal melanoma has a long history, the first successfully treated case being reported by Deutschmann 1 . In 1913, he applied mesothorium, a natural isotope, to an only eye, resulting 7 months later in flat tumor scar. In 1929, Foster-Moore was the first to insert radon needles (“Curie-puncture”) into the choroidal melanoma, resulting in its regression 1 , 2 . In 1948, it was Stallard who developed episcleral radio-active plaques, using the synthetic isotope, Cobalt-60, emitting a bidirectional, strong gamma irradiation 1 , 2 . Subsequently, since 1968 and 1975 respectively, Ruthenium-106 and Iodine-125 plaques were introduced, offering a more localized irradiation, and reduced exposure to both the orbital structures of the patient and the hands of the medical staff 1 , 3 . In 1975, Gragoudas treated the first patient with external beam proton therapy, providing a conservative alternative to patients with tumors inaccessible to brachytherapy, as well as an excellent local tumor control 1 , 4 .

In 2006, the COMS study gave an answer to a long-standing controversy, by demonstrating that the metastatic risk was not higher in uveal melanoma patients treated with conservative (Iodine-125) radiation therapy than in those managed with enucleation 5 . However, in 2016, the Ophthalmic Oncology Task Force insisted on the importance of local tumor control, the hazard ratio (HR) of developing metastases being 6.28 in case of a local recurrence following conservative treatment 6 .

We report the exceptional case of a recurrent choroidal melanoma, 51,5 years following Cobalt-60 brachytherapy, which we re-treated with conservative proton therapy. One year later, the tumor has regressed and the patient has no metastases.

## Case Report

**Fig. 1:**
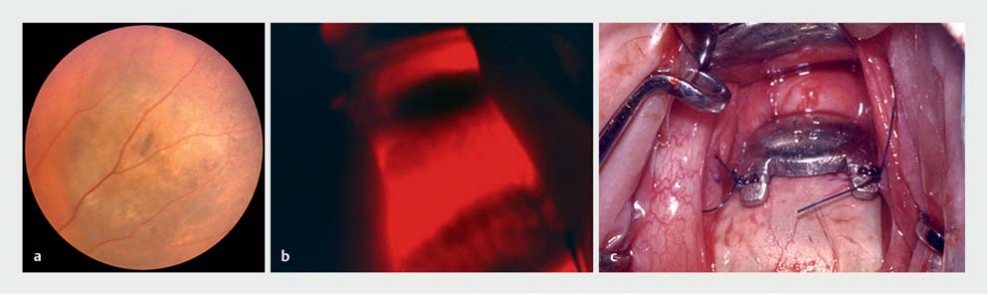
Initial presentation and management in the early 70’s. **a** Asymptomatic pigmented tumor in the nasal superior equatorial choroid (RE). **b** Perioperative transillumination locates the pigmented intraocular lesion. **c** Cobalt-60 plaque (CKA2) covering the sclera at the base of the choroidal melanoma.

In the early 70’s, a 36-year-old male was diagnosed with an asymptomatic pigmented tumor in the nasal superior equatorial choroid of his right eye (RE, Fig. 1 a ), measuring 7 × 7 mm in basal diameter and 3.5 mm in thickness on B-scan ultrasonography, and associated with a limited secondary serous retinal detachment. Snellen best corrected visual acuity (BCVA) was 1.2 (RE) and 1.0 (LE), and ocular findings were otherwise within normal limits. A systemic work-up was negative for metastases.

During the ensuing intervention, transillumination permitted to locate the intra-ocular lesion ( Fig. 1 b ), followed by a diagnostic radioactive phosphorus P-32 uptake test which resulted positive, confirming the diagnosis of a choroidal melanoma. In consequence, a Cobalt-60, 7.5 mm plaque (CKA2) was applied to the sclera ( Fig. 1 c ) for nine days, delivering 69 Gray at the tumor apex.

**Fig. 2:**
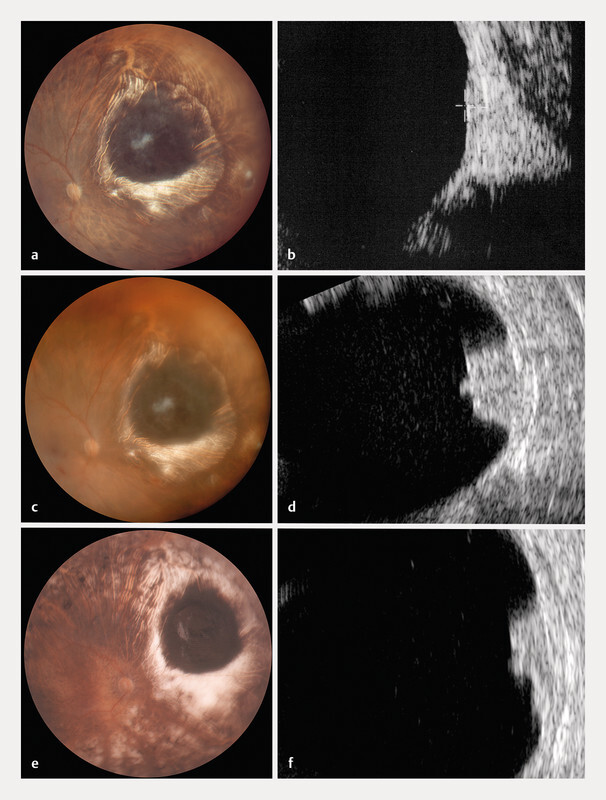
Follow-up color fundus pictures and B-scan ultrasonography. **a** , **b** 37 years later: apparent local tumor control. **c** , **d** 51.5 years later: a recurrent vitreous hemorrhage leads to the diagnosis of a local recurrence, with a significant increase in tumor thickness. **e** , **f** One year following proton therapy, the recurrent melanoma has regressed.

Follow-up consisted of yearly control examinations, which were progressively spaced out to every five years with intermediate check-ups by the local ophthalmologist. In the late 90’s, the patient underwent coronary bypass surgery because of a severe heart-attack and had anti-platelet therapy ever since. At two and 28 years, a Xenon and Argon laser photo-coagulation respectively was performed to treat a secondary neovascular subretinal membrane at the apex of the tumor, associated with retinal hemorrhages. The latter procedure was immediately complicated by floaters, signs of a mild vitreous hemorrhage, which spontaneously reabsorbed, though transiently recurred nearly 6 and 7 years later. Nine years later, i.e. 37 years following brachytherapy, another, this time persistent, vitreous hemorrhage recurred, for which angiography could not identify an apparent origin, the already treated neovascular membrane appearing completely fibrotic at the tumor apex, and which was successfully managed with posterior vitrectomy and pan fundus endolaser photocoagulation ( Fig. 2 a ). At that time, residual tumor thickness was 1.3 mm ( Fig. 2 b ). A year later, bilateral phacoemulsification was performed.

**Fig. 3:**
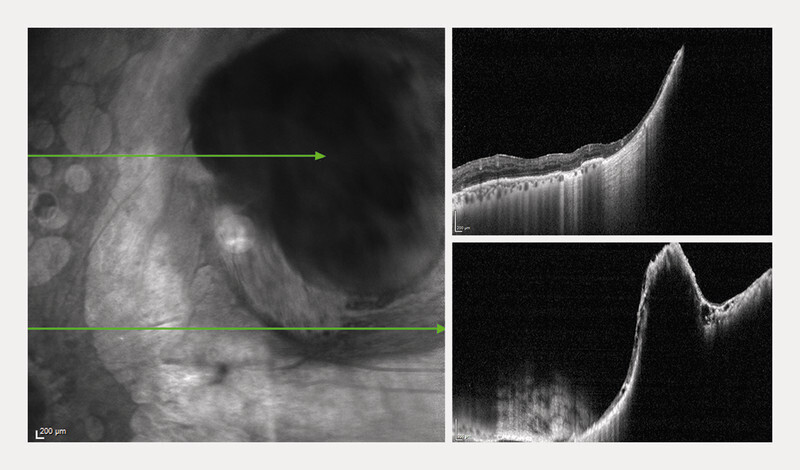
OCT B-scan examining the borders of the recurrent tumor ( **a** ), emerging from the choroid and covered by an atrophic retina ( **b** ), with a small rupture of Bruchʼs membrane close to its inferior margin ( **c** ).

**Fig. 4:**
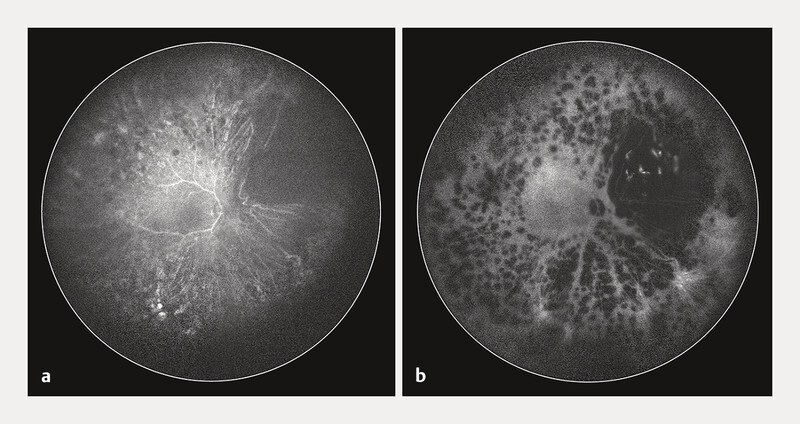
Panoramic HRA 150° fundus angiography of the recurrent melanoma (RE): **a** On fluorescein angiography, no secondary neovascular membrane can be identified. **b** ICG angiography allows to visualize dilated intralesional vessels, discarding the differential diagnosis of a blood clot.

However, 51 and a half years after Cobalt-60 plaque therapy, the patient was referred to us because of a recurrent and persistent vitreous hemorrhage, with a six monthsʼ history of floaters and vision loss, as well as an increase in tumor thickness up to 4.0 mm. On examination, BCVA was 0.5 (RE). Despite the presence of red blood cells in both the anterior chamber and the vitreous, the fundus tumor could be visualized and its margins seemed stable. There were no signs of any intra- or subretinal hemorrhages ( Fig. 2 c ). An OCT B-scan permitted to observe the borders of a solid mass, emerging from the choroid and covered by an atrophic retina ( Fig. 3 ). On fluorescein angiography, there were no signs of a secondary neovascular membrane ( Fig. 4 a ), and the presence of dilated intra-lesional vessels on ICG-angiography discarded the differential diagnosis of a blood clot ( Fig. 4 b ). Also, on B-scan ultrasonography, tumor thickness had not started to regress and was still measured at 4.0 mm ( Fig. 2 d ), compatible with the diagnosis of a recurrent choroidal melanoma. A systemic work-up did not reveal metastases, but the presence of a severe heart failure was a contra-indication for general anesthesia.

As BCVA in this RE was still useful, a second conservative radiation therapy was proposed, this time with protons (60 CGE in 4 fractions), the tantalum clips being placed under loco-regional anesthesia. On control examination one year later, BCVA had remained stable at 0.5 (RE). The tumor scar ( Fig. 2 e ) had regressed to a residual thickness of 2.9 mm ( Fig. 2 f ). There was no evidence of systemic metastases.

## Discussion

We describe the case of an exceptionally late local recurrence of a choroidal melanoma, 51 years and 6 months following Cobalt-60 plaque brachytherapy, which, to our knowledge, is the longest interval reported in the literature.

Several aspects of this case deserve consideration. First, with regard to the etiology of this recurrence, presumably related to an underdosage of the irradiation delivered to the tumor apex (69 Gy), which turned out to be below the recommendations established later, suggesting that an apex dose of ≥ 90 Gy 7 or even ≥ 130 Gy 2 was required to prevent recurrence.

Second, this case highlights the potential for very late tumor reactivation, which may have been underestimated. Most local recurrences occur within the first 5 years after brachytherapy 3 , with a range following Cobalt-60 plaque therapy between 3 months and 10.2 years 8 , and the longest interval being reported, to our knowledge, by Shields i.e. 15 years and 8 months 9 . Our case emphasizes the critical need for lifelong surveillance following conservative radiation therapy for uveal melanoma.

Third, this case also illustrates that local recurrence does not necessarily imply rapid appearance of metastatic spread. We speculate that one must differentiate this risk for early local recurrences from that of the late ones.

Fourth, this case illustrates the feasibility of salvage proton therapy 4 , 10 , even in elderly patients with significant comorbidities, where radiation therapy proved to be the lesser aggressive approach and not only allowed tumor regression, but also preservation of BCVA at 0.5, confirming that a local recurrence does not necessarily imply secondary enucleation.
